# Mice overexpressing growth hormone exhibit increased skeletal muscle myostatin and MuRF1 with attenuation of muscle mass

**DOI:** 10.1186/s13395-017-0133-y

**Published:** 2017-09-04

**Authors:** Leslie A. Consitt, Alicson Saneda, Gunjan Saxena, Edward O. List, John J. Kopchick

**Affiliations:** 1 Department of Biomedical Sciences, Ohio University, 228 Irvine Hall, Athens, Ohio 45701 USA; 2 Ohio Musculoskeletal and Neurological Institute, Ohio University, Athens, Ohio 45701 USA; 3 Diabetes Institute, Ohio University, Athens, Ohio 45701 USA; 4 Department of Biological Sciences, Ohio University, Athens, Ohio 45701 USA; 5 Edison Biotechnology Institute, Ohio University, Athens, OH 45701 USA

## Abstract

**Background:**

In contrast to the acute effects of growth hormone (GH) on skeletal muscle protein synthesis, long-term GH treatment appears to have negligible effects on muscle mass. Despite this knowledge, little is known regarding the chronic effects of GH on skeletal muscle protein synthesis and atrophy signaling pathways. The purpose of this study was to determine if protein synthesis pathways are attenuated and/or muscle atrophy intracellular signaling pathways are altered in the skeletal muscle of transgenic bovine GH (bGH) mice.

**Methods:**

The gastrocnemius and soleus from 5-month-old male bGH mice (*n* = 9) and wild type (WT) controls (*n* = 9) were harvested and analyzed for proteins involved in the protein synthesis (Akt/mTOR), growth and proliferation (MAPK), and muscle atrophy (MuRF1 and myostatin) pathways.

**Results:**

Total body mass was significantly increased in bGH mice compared to WT controls (49%, *P* < 0.0001). When expressed relative to total body mass, the gastrocnemius (− 28%, *P* < 0.0001), but not the soleus, was significantly lower in mice overexpressing GH, compared to controls. Transgenic bGH mice had elevated phosphorylation levels of protein kinase b (Akt1), 4E-binding protein 1 (4E-BP1), p70 S6 kinase, p42/44, and p38 (*P* < 0.05) compared to WT littermates. Mature myostatin (26 kDa), premature myostatin (52 kDa), and activin receptor type IIB (AcvR2B) protein levels were increased in bGH mice (*P* < 0.05), along with elevated phosphorylation levels of mothers against decapentaplegic homolog (Smad2) (59%, *P* < 0.0001). Mice overexpressing GH had increased MuRF1 expression (30%, *P* < 0.05) and insulin receptor substrate 1 (IRS1) serine phosphorylation (44%, *P* < 0.05) in the gastrocnemius, but not the soleus, when compared to controls.

**Conclusions:**

These findings demonstrate that chronic elevations in circulating GH have a critical impact on signaling pathways involved in skeletal muscle protein synthesis and atrophy, and suggest that MuRF1, myostatin, and IRS1 serine phosphorylation may act to inhibit exaggerated glycolytic muscle growth, in environments of chronic GH/IGF-1 excess.

## Background

Skeletal muscle mass is determined by the net balance between intramuscular pathways responsible for protein synthesis and atrophy. The decline in muscle mass is associated with reduced quality of life [1], as well as, a number of health related conditions including, aging [2], cancer [3], chronic obstructive pulmonary disease (COPD) [4, 5], cystic fibrosis [6], and acquired immune deficiency syndrome (AIDS) [6]. Despite this knowledge, the cellular mechanisms responsible for regulating muscle mass, especially during chronic conditions, remain unclear.

Growth hormone (GH), a protein secreted from the anterior pituitary gland, is an anabolic hormone that plays a critical role in growth [7] and metabolism [8]. While many of the growth promoting properties of GH in skeletal muscle have been attributed to the indirect effects of insulin-like growth factor-1 (IGF-1) [9], acute GH infusion studies have provided compelling evidence that this hormone also has a direct role in stimulating skeletal muscle protein synthesis [10, 11]. GH binds to its receptor leading to the recruitment and phosphorylation of Janus kinase 2 (JAK2) and its most recognized downstream target, signal transducer and activator of transcription 5 (STAT5) [12, 13]. Similar to IGF-1, GH stimulates the IRS1/Akt [14–16] and mitogen-activated protein kinase (MAPK) [16, 17] pathways which are thought to be the main pathways contributing to GH/IGF-1-induced muscle hypertrophy. The activation of Akt1 is a primary event in regulating skeletal muscle development since it controls the phosphorylation of a number of substrates involved in protein synthesis including mechanistic target of rapamycin (mTOR) (and its downstream targets 4E-binding protein 1 (4E-BP1) and p70S6 kinase) and glycogen synthase kinase 3β (GSK3β), as well as, the inhibition of protein degradation via the forkhead transcription factor (FOXO) pathway. The MAPK signaling cascade, especially the p42/p44 and p38 pathways, are also known to have an important role in skeletal muscle proliferation and differentiation [18–20].

Declines in circulating GH and IGF-1 often parallel decreases in skeletal muscle mass, making GH supplementation a potentially attractive treatment for conditions of GH deficiency. However, unlike the short-term effects of GH on skeletal muscle protein synthesis [21], the majority of research examining the effects of chronic GH exposure have been negligible. For example, increases in skeletal muscle mass were reported to plateau after only 16 weeks of GH replacement therapy in GH-deficient adults [22] and no gains in skeletal muscle hypertrophy were achieved in healthy, elderly men after 10–12 weeks of GH supplementation [23, 24]. These findings are further supported by studies using adults with acromegaly, a condition with excess circulating levels of GH and IGF-I. Patients with active acromegaly experience organomegaly [25]; however, these individuals either have similar skeletal muscle mass as healthy controls [26] or evidence of muscle atrophy, especially in type 2 fibers [27, 28].

The lack of muscle hypertrophy during conditions of chronic GH excess suggest the IRS1/Akt and/or MAPK pathways become desensitized, or alternative pathways involved in muscle atrophy become upregulated. Muscle RING factor 1 (MuRF1) and myostatin are two candidates that could play a role in GH-induced muscle atrophy since both are preferentially expressed in type 2 muscle fibers and believed to play a critical role in fast-twitch muscle atrophy [29, 30]. MuRF1 is an E3 ubiquitin ligase, responsible for proteasomal degradation [31], and can act independent of the myostatin pathway to induce muscle atrophy. Myostatin, a member of the transforming growth factor-β (TGF-β) superfamily is synthesized in skeletal muscle [32] and promotes muscle wasting through stimulation of its canonical Smad2/3 pathway. In cardiac tissue, myostatin has been suggested to act as a chalone [33, 34], a protein secreted by tissue to provide a negative feedback mechanism to control tissue size [35]. Preliminary research indicates that GH signaling may play a role in regulating myostatin expression [36]; however, no known studies have examined the Smad2/3 pathway in response to GH excess.

Bovine GH (bGH) mice have frequently been used as a model to study the effects of chronic GH exposure [37–39]. Similar to patients with acromegaly, these mice have increased linear growth [39], decreased body fat [38, 39], and are insulin resistant [37, 40, 41]. Of particular interest, organs including the liver, kidney, heart, and lungs experience exaggerated growth in bGH mice even when expressed relative to total body weight [39], whereas relative skeletal muscle mass does not differ from control mice [42]. Further, absolute grip strength has been reported to be similar between mice overexpressing GH and controls, and decreases in strength have been documented in bGH mice when expressed relative to body weight, suggesting chronic GH excess may produce less efficient muscle [43]. These findings support a tissue-specific negative feedback mechanism in skeletal muscle during conditions of GH excess. The main objectives of the current study was to (1) determine if key intracellular signaling pathways responsible for protein synthesis are attenuated in the skeletal muscle of bGH mice and (2) determine if MuRF1 and/or the myostatin pathway are upregulated in bGH mice, suggesting these protein act as a skeletal muscle chalone in conditions of chronic GH/IGF1 exposure.

## Methods

### Animals

Male bGH transgenic (*n* = 9) and wild type littermate controls (*n* = 9) were generated as previously described [38, 39]. Briefly, bGH mice were generated by a pronuclear injection into a C57BL/6J embryo with the bGH complementary DNA (cDNA) fused to the mouse metallothionein transcriptional regulatory element. Mice were screened for the bGH gene using polymerase chain reaction (PCR), and bGH males were bred to non-transgenic (NT) females to further propagate the line. Successive generations were bred and screened in a similar manner. Mice were housed 2–4 mice per cage in the temperature-controlled (23 °C) vivarium and exposed to 14-h light/10-h dark cycle. All mice were allowed ab libitum access to water and food (ProLab RMH 3000; PMI Nutrition International). At 5 months of age, mice were euthanized by CO_2_ inhalation and the gastrocnemius and soleus muscles were collected. Mice were fasted for 12 h prior to euthanasia. All procedures performed with the mice were approved by the Institutional Animal Care and Use Committee at Ohio University and are in accordance with all standards set forth by federal, state, and local authorities.

### Western blot procedure

Skeletal muscle was homogenized, and protein content was determined as previously described [44, 45]. Muscle lysate (20–30 μg cellular protein) was separated by sodium dodecyl sulfate polyacrylamide gel electrophoresis (SDS-PAGE), electrotransferred onto polyvinylidene difluoride membranes (Millipore, Billerica, MA) and probed overnight with Cell Signaling (Beverly, MA) antibodies for JAK2 (Tyr1007/1008), JAK2 total, STAT5 (Tyr694), STAT5 total, Akt1 (Ser473), Akt2 (Ser474), Akt1 total, Akt2 total, AS160 (Ser588), AS160 (Ser666), AS160 total, TBC1D1 (Thr590), TBC1D1 (Ser700), TBC1D1 total, insulin receptor β, IRS1 (Ser307), mTOR (Ser2448), mTOR total, p70 S6 kinase (Thr389), p70 S6 kinase total, 4E-BP1 (Thr37/46), 4E-BP1 total, p38 (Thr180/Tyr182), p38 total, p44/42 (Thr202/Tyr204), p44/42 total, JNK (Thr183/Tyr185), JNK total, GSK-3β (Ser9), GSK3α/β total, Fox01 (Thr24)/Fox03a (Thr32), FOXO1 total, glyceraldehyde 3-phosphate dehydrogenase (GAPDH), Santa Cruz Biotechnology (Santa Cruz, CA) antibodies for IRS1 total, myostatin, and muscle RING factor 1 (MuRF1), Developmental Studies Hybridoma Bank (University of Iowa) antibodies for myosin heavy chain (MHC) I and MHC IIb, as well as, an Abcam (Cambridge, MA) antibody for Activin Receptor Type IIB. Proteins were visualized by horseradish peroxidase-conjugated IgG antibodies (Santa Cruz, CA) and Amersham ECL Prime Western Blotting Detection Reagent (GE Healthcare Life Sciences), then exposed to X-ray film. Band densitometry analysis was determined using ImageJ software (NIH). Samples were normalized to GADH levels, and a control sample was resolved on each gel. Additionally, phosphorylation levels were normalized to their corresponding total protein after membranes were stripped, as previously reported [44].

### Statistics

Analyses were performed using SPSS version 21.0 software (SPSS Inc., Chicago, IL). Comparisons between bGH mice and WT controls were analyzed using unpaired *t* test comparisons. Pearson correlation coefficients were used to determine relationships between proteins. Data are presented as means ± SEM unless otherwise noted. Statistical significance was defined as *P* < 0.05.

## Results

### Body weight and gastrocnemius mass

Total body mass was significantly increased in bGH mice compared to WT controls (49%, *P* < 0.0001, Table 1). Mice overexpressing GH had increased soleus muscle mass (44%, *P* < 0.0001, Table 1) and a tendency for increased gastrocnemius muscle mass (*P* = 0.06, Table 1) compared to controls; however, the gastrocnemius was lower (− 28%, *P* < 0.0001, Table 1) and the soleus did not differ (*P* = 0.44) when expressed relative to total body weight.

**Table 1:** Mass of whole body, gastrocnemius, and soleus in bGH and WT mice

	WT mice	bGH mice
Whole body mass (g)	30.44 ± 1.78	45.22 ± 2.91 *
Gastrocnemius mass (g)	0.310 ± 0.019	0.332 ± 0.027 †
Gastrocnemius/body mass (g)	0.0102 ± 0.0007	0.0073 ± 0.0004 *
Soleus mass (g)	0.017 ± 0.002	0.024 ± 0.003 *
Soleus/body mass (g)	0.0006 ± 0.00006	0.0005 ± 0.00004

Mass of both gastrocnemius and soleus. Data are presented mean ± SD

*WT* wild type control mice (*n* = 9), *bGH* bovine growth hormone mice (*n* = 9)

*P < 0.0001 vs. WT mice, † *P* = 0.06 vs. WT mice

### Skeletal muscle JAK2-STAT5 pathway

**Fig. 1:**
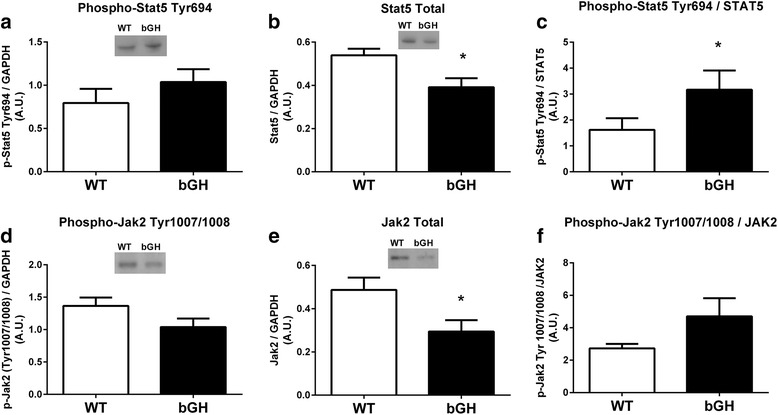
JAK2/STAT5 signaling in the gastrocnemius of bGH (*n* = 9) and WT (*n* = 9) male mice. STAT5 tyrosine phosphorylation of site 694 (**a**) STAT5 total protein content (**b**) and STAT5 phosphorylation (Tyr 694) relative to STAT5 total protein (**c**). JAK2 tyrosine phosphorylation of site 1007/1008 (**d**) JAK2 total protein content (**e**) and JAK2 phosphorylation (Tyr 1007/1008) relative to JAK2 total protein (**f**). Values in A.U. (arbitrary units) were normalized to a control sample run on each blot and then presented relative to GAPDH. Data are expressed as mean ± SEM. * *P* < 0.05 vs. WT control mice

Tyrosine phosphorylation of STAT5 was increased in the gastrocnemius of bGH mice compared to WT controls only when expressed relative to total protein (96%, *P* < 0.05, Fig. 1c). JAK2 phosphorylation relative to total JAK2 protein in the gastrocnemius did not differ between mice overexpressing GH and controls (*P* = 0.12, Fig. 1f). Total STAT5 and JAK2 protein were significantly lower in the gastrocnemius of bGH mice (− 38%, Fig. 1b and − 40%, Fig. 1e, respectively, *P* < 0.05). Due to limited soleus tissue, JAK2 and STAT5 analyses were only completed in a subset of WT (*n* = 5) and bGH (*n* = 4) mice; however, similar trends were observed in the soleus as in the gastrocnemius.

### Skeletal muscle Akt/mTOR pathway

**Fig. 2:**
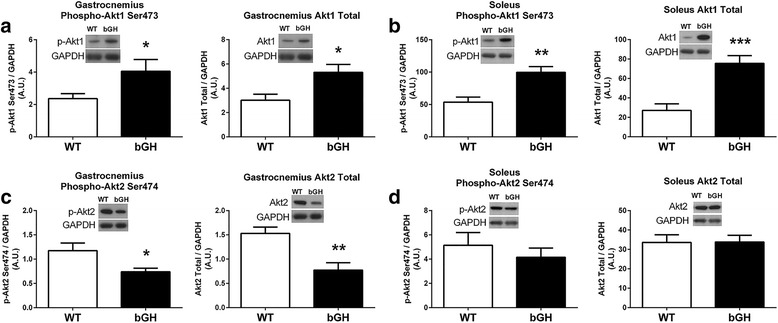
Akt1 and Akt2 phosphorylation and total protein content in the gastrocnemius and soleus of bGH (*n* = 9) and WT (*n* = 9) male mice. Phosphorylation of Akt1 Serine 473 and Akt1 total protein in gastrocnemius of WT and bGH mice (**a**). Soleus phosphorylation of Akt1 Serine 473 and total protein in WT and bGH mice (**b**). Phosphorylation of Akt2 Serine 474 and Akt2 total protein in the gastrocnemius of bGH mice and WT controls (**c**). Soleus phosphorylation of Akt2 Serine 474 and total protein in the gastrocnemius of bGH mice and WT controls (**d**). Values in A.U. were normalized to a control sample run on each blot and then presented relative to GAPDH. Data are expressed as mean ± SEM. * *P* < 0.05, ** *P* < 0.005, ***P < 0.0005 vs. WT control mice. gastrocnemius, soleus

Skeletal muscle Akt1 phosphorylation on site Ser473 was 72% higher in the gastrocnemius of bGH compared to WT mice (*P* < 0.05, Fig. 2a). Similarly, the soleus of bGH mice had increased phosphorylation levels of Akt1 Ser473 (86%, *P* < 0.005, Fig. 2b) compared to controls. The gastrocnemius of bGH had decreased Akt2 phosphorylation on site Ser474 compared to WT mice (− 37%, *P* < 0.05, Fig. 2c), whereas no significant differences in Akt2 serine phosphorylation were detected between the two groups of mice in the soleus (Fig. 2d). Total Akt1 was 76 and 179% higher in the gastrocnemius (*P* < 0.05, Fig. 2a) and soleus (*P* < 0.0005, Fig. 2b), respectively, of the bGH mice compared to WT controls. Total Akt2 was 50% lower (*P* < 0.005, Fig. 2c) in the gastrocnemius of bGH mice compared to their WT counterparts, whereas Akt2 protein content did not differ in the soleus (Fig. 2d). When phosphorylation was normalized to the corresponding Akt isoform, no differences existed between the two groups of mice, with the exception of the soleus of WT mice that had an increase in serine 473 phosphorylation on Akt1 relative to total protein (44%, *P* < 0.05).

**Fig. 3:**
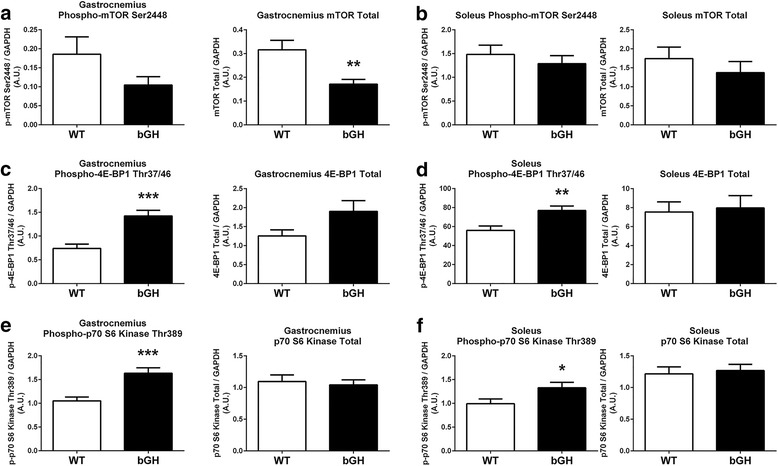
Phosphorylation and total protein involved in protein synthesis in the gastrocnemius and soleus of bGH (*n* = 9) and WT (*n* = 9) mice. mTOR phosphorylation and mTOR total protein in the gastrocnemius (**a**) and soleus (**b**) of WT and bGH mice. Phosphorylation of 4E-BP1 and 4E-BP1 total protein in the gastrocnemius (**c**) and soleus (**d**) of WT and bGH mice. p70 S6 kinase phosphorylation and p70 S6 kinase total protein in the gastrocnemius (**e**) and soleus (**f**) of WT and bGH mice. Values in A.U. were normalized to a control sample run on each blot and then presented relative to GAPDH. Data are expressed as mean ± SEM. * *P* < 0.05, ** *P* < 0.01, *** *P* < 0.001 vs. WT control mice

mTOR protein was significantly depressed in the gastrocnemius of bGH mice compared to WT controls (− 85%, *P* < 0.01, Fig. 3a) with no statistical differences noted in the phosphorylation of mTOR in the gastrocnemius (*P* = 0.13, Fig. 3a) or soleus (*P* = 0.45, Fig. 3b). Phosphorylation of 4EBP1 and p70 kinase were significantly increased in the gastrocnemius (92 and 55%, respectively, *P* < 0.001, Fig. 3c, e) and soleus (37%, *P* < 0.01 and 34%, *P* < 0.05, respectively, Fig. 3d, f) of bGH mice, with no differences in total protein (Fig. 3c–f). GSK3β and FOXO1 phosphorylation, as well as, total protein did not differ between bGH and WT mice (not shown). When phosphorylated proteins were normalized to respective total protein, only p70 S6 kinase phosphorylation levels in the gastrocnemius were significantly elevated in the bGH mice compared to WT controls (78%, *P* < 0.005, not shown).

### Skeletal muscle MAPK pathway

**Fig. 4:**
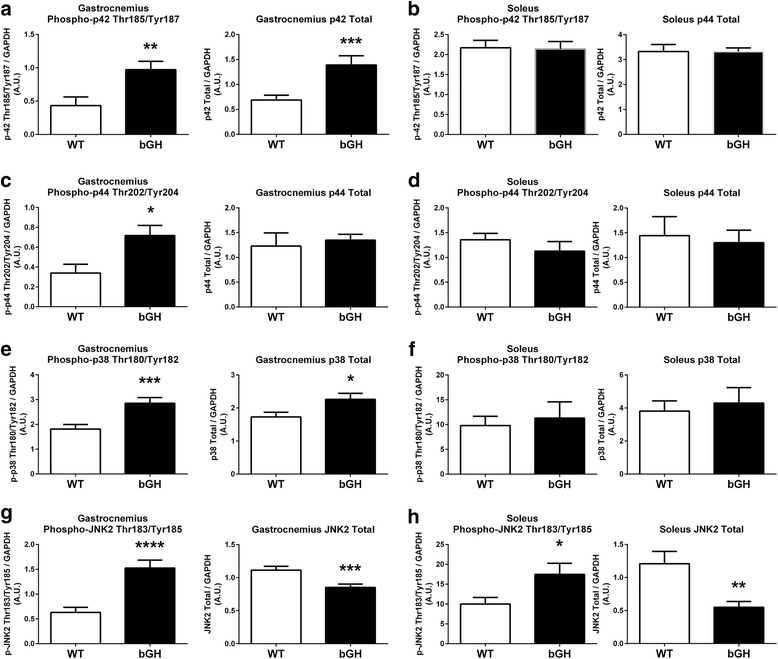
MAPK phosphorylation and protein content in gastrocnemius and soleus of bGH and WT mice. Phosphorylation of p42 and total p42 in the gastrocnemius (**a**) and the soleus (**b**) of WT and bGH mice. p44 phosphorylation and p44 total protein in the gastrocnemius (**c**) and soleus (**d**) of WT and bGH mice. Phosphorylation of p38 and total p38 in the gastrocnemius (**e**) and the soleus (**f**) of WT and bGH mice JNK2 phosphorylation and JNK2 total protein in the gastrocnemius (**g**) and the soleus (**h**) of WT and bGH mice. Values in A.U. were normalized to a control sample run on each blot and then presented relative to GAPDH. Data are expressed as mean ± SEM. * *P* < 0.05, **P < 0.01, ***P < 0.005, ****P < 0.0005 vs WT control mice. bGH (*n* = 9) and WT (*n* = 9) for each protein with the exception of (**c**) gastrocnemius p44 phosphorylation and p44 total protein (*n* = 7 in each group)

**Fig. 5:**
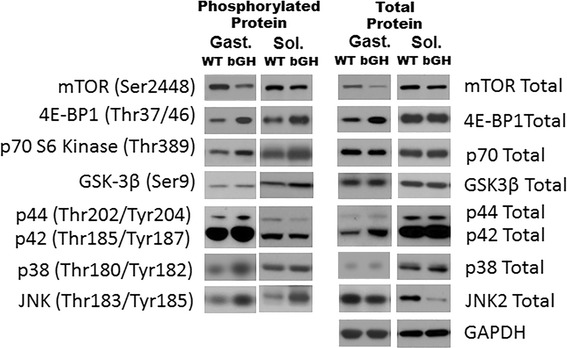
Representative blots for the mTOR (Fig. 3) and the MAPK pathway (Fig. 4) in the gastrocnemius and soleus of WT and bGH mice. Gast gastrocnemius, Sol soleus

Phosphorylation of p42 and p44 were increased in the gastrocnemius of bGH mice compared to WT littermates (124%, *P* < 0.01, Fig. 4a and 111%, *P* < 0.05, Fig. 4c, respectively). Gastrocnemius phosphorylation of p38 was increased 57% (*P* < 0.005, Fig. 4e) in the bGH mice compared to controls. Phosphorylation of JNK2 was increased in the bGH mice 142% (*P* < 0.0005, Fig. 4g) and 75% (*P* < 0.05, Fig. 4h) in the gastrocnemius and soleus, respectively. Total p42 and p38 were increased in the gastrocnemius (102%, *P* < 0.005, Fig. 4a, and 31%, *P* < 0.05, Fig. 4e, respectively) of bGH mice, whereas total JNK2 was decreased − 23% in the gastrocnemius (*P* < 0.005, Fig. 4g) and − 54% in the soleus (*P* < 0.01, Fig. 4h) of bGH mice compared to control mice. When phosphorylated proteins were normalized to respective total protein, only JNK2 phosphorylation was significantly increased in the gastrocnemius (220%, *P* < 0.0001, not shown) and soleus (510%, *P* < 0.05, not shown) (blots, Fig. 5).

### Skeletal muscle MuRF1 protein

**Fig. 6:**
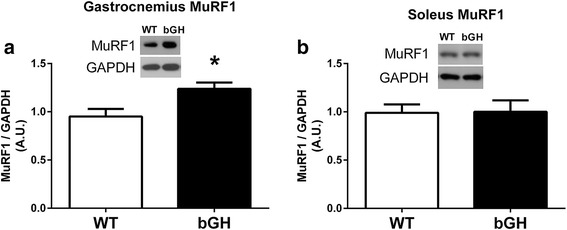
Skeletal muscle MuRF1 protein expression in bGH (*n* = 9) and WT (*n* = 9) mice. Gastrocnemius (**a**) and soleus (**b**) MuRF1 protein expression in bGH and WT mice. Values in A.U. were normalized to a control sample run on each blot and then presented relative to GAPDH. Data are expressed as mean ± SEM. * *P* < 0.05 vs WT control mice

MuRF1 protein content was elevated in the gastrocnemius (30%, *P* < 0.05, Fig. 6a), but not the soleus (*P* = 0.94), of bGH mice compared to their WT counterparts. Gastrocnemius MuRF1 was not significantly associated with gastrocnemius mass relative to body weight (*r* = −0.43, *P* = 0.08).

### Skeletal muscle myostatin and Smad2/3 pathway

**Fig. 7:**
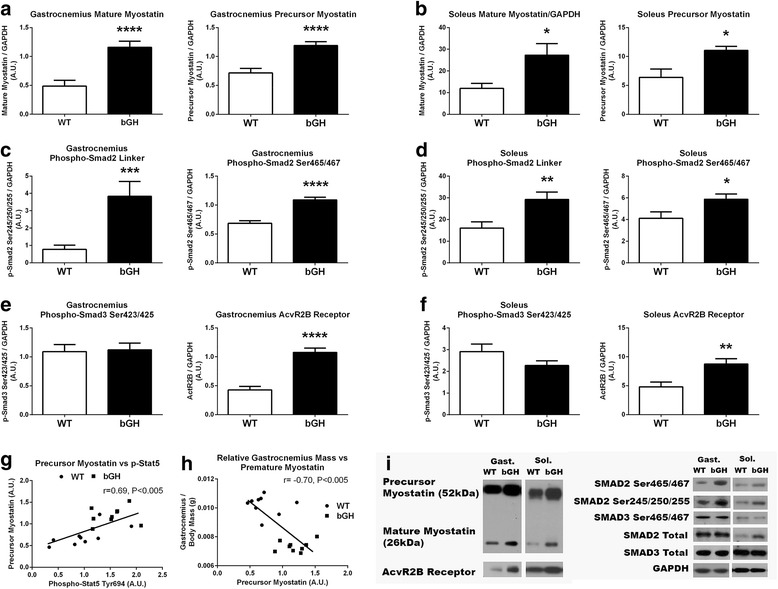
Myostatin signaling in the gastrocnemius and soleus of bGH (*n* = 9) and WT (*n* = 9) mice. Mature and precursor myostatin protein content in the gastrocnemius (**a**) and soleus (**b**) of WT and bGH mice. Phosphorylation of Smad2 (Ser245/250/255) and (Ser465/467) in the gastrocnemius (**c**) and soleus (**d**) of WT and bGH mice. Phosphorylation of Smad3 (Ser 423/425) and AcvR2B total protein in the gastrocnemius (**e**) and soleus (**f**) of WT and bGH mice. Relationship between Precursor Myostatin and STAT5 phosphorylation in gastrocnemius of bGH and WT skeletal muscle (**g**). Relationship between relative gastrocnemius mass and precursor myostatin in the gastrocnemius of bGH and WT skeletal muscle (**h**). Representative blots in the gastrocnemius and soleus of WT and bGH mice (**i**). Values in A.U. were normalized to a control sample run on each blot and then presented relative to GAPDH. Data are expressed as mean ± SEM. * *P* < 0.05, **P < 0.01, ***P < 0.005 ****P < 0.0005 vs. WT control mice. Gast, gastrocnemius; Sol, soleus

The gastrocnemius and soleus of bGH mice had higher levels of mature myostatin (138%, *P* < 0.0005, Fig. 7a and 128%, *P* < 0.05, Fig. 7b, respectively), precursor myostatin (66%, *P* < 0.0005, Fig. 7a and 73%, *P* < 0.05, Fig. 7b, respectively), and the myostatin receptor, activin receptor type IIB (AcvR2B) (152%, *P* < 0.0005, Fig. 7e and 82%, *P* < 0.01, Fig. 7f) compared to WT mice. Phosphorylation of STAT5 was significantly associated with precursor myostatin (*r* = 0.69, *P* < 0.005, Fig. 7g) in the gastrocnemius of the whole group. Gastrocnemius premature myostatin (*r* = −0.70, *P* < 0.005, Fig. 7h) and mature myostatin (*r* = − 0.69, *P* < 0.005, not shown) were negatively associated with the gastrocnemius mass relative to body weight. Phosphorylation of mothers against decapentaplegic homolog (Smad2) on serine site 465/467 was increased in the gastrocnemius (59%, *P* < 0.0005) and the soleus (43%, *P* < 0.05) of the bGH compared to WT mice. The gastrocnemius and soleus of bGH mice had a 400% (*P* < 0.005, Fig. 6e) and 82% (*P* < 0.01) increase in phosphorylation levels of Smad2 in the linker region (serine sites 245/250/255) compared to their WT controls. Phosphorylation of Smad3 on serine site 423/425 in the gastrocnemius and soleus did not differ between the two groups of mice. In the whole group, mature myostatin was positively associated with phosphorylation on the Smad2 serine site 423/425 in the soleus (*r* = 0.47, *P* < 0.05, not shown) and the gastrocnemius (*r* = 0.33, *P* < 0.05, not shown), but not on Smad3 serine site 423/425 (*P* > 0.22) or the Smad2 linker region (*P* > 0.30). There were no differences in Smad2 or Smad3 (blots, Fig. 7i) total protein between bGH and WT mice.

### Myosin heavy chain (MHC) I and IIb

**Fig. 8:**
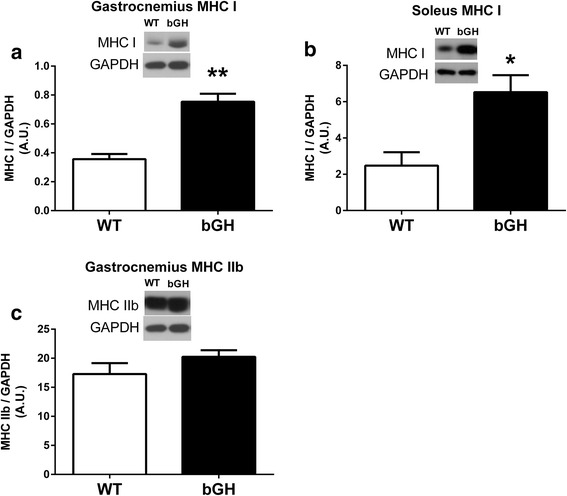
Skeletal muscle MHC in WT (*n* = 9) and bGH (*n* = 9) mice. Gastrocnemius (**a**) and soleus (**b**) MHC I protein expression in WT and bGH mice. Gastrocnemius (**c**) MHC IIb in WT and bGH mice. Values in A.U. were normalized to a control sample run on each blot and then presented relative to GAPDH. Data are expressed as mean ± SEM. *P < 0.05, ** *P* < 0.0001 vs. WT control mice. Gast gastrocnemius, Sol soleus

MHC I was increased 112% in the gastrocnemius (*P* < 0.0001, Fig. 8a) and 163% in the soleus (*P* < 0.01, *n* = 4 WT and *n* = 5 bGH, Fig. 8b) of bGH mice compared to the WT mice. Gastrocnemius MHC IIb did not differ between the two groups of mice (*P* = 0.20, Fig. 8c). MHC IIb was undetectable in the soleus muscle of both the bGH and WT mice.

### Insulin signaling intermediates

Gastrocnemius and soleus insulin receptor beta levels were significantly reduced in bGH mice compared to WT controls (− 47%, *P* < 0.0001 and − 41% *P* < 0.05, respectively, Table 2). Phosphorylation on insulin receptor substrate 1 (IRS1) serine site 307 was significantly increased in the gastrocnemius (44%, *P* < 0.05, Table 2), but not soleus (*P* = 0.10) of bGH mice. There was a trend for IRS1 total protein to be increased in the gastrocnemius of bGH compared to controls (62%, *P* = 0.06, Fig. 7b), but not the soleus (*P* = 0.30).

**Table 2:** Insulin signaling intermediates

	Gastrocnemius		Soleus	
	WT mice (*n* = 9)	bGH mice (*n* = 9)	WT mice (*n* = 9)	bGH mice (*n* = 9)
Insulin receptor β (AU)	1.50 ± 0.01	0.80 ± 0.05 ***	2.08 ± 0.24	1.23 ± 0.18 *
p-IRS-1Ser307 (AU)	0.76 ± 0.07	1.09 ± 0.14 *	0.84 ± 0.23	1.46 ± 0.25
IRS1 total (AU)	1.59 ± 0.21	2.57 ± 0.44	1.57 ± 0.33	1.10 ± 0.31
p-AS160^Ser588^ (AU)	0.40 ± 0.03	0.49 ± 0.05	1.67 ± 0.40	1.85 ± 0.29
p-AS160^Ser666^ (AU)	1.08 ± 0.10	1.89 ± 0.16 **	1.76 ± 0.31	2.11 ± 0.34
p-TBC1D1^Thr590^ (AU)	0.23 ± 0.05	0.29 ± 0.04	1.71 ± 0.19	1.18 ± 0.16 *
p-TBC1D1^Ser700^ (AU)	0.28 ± 0.04	0.42 ± 0.08	1.12 ± 0.19	0.81 ± 0.22
AS160 (AU)	0.50 ± 0.04	0.74 ± 0.08 *	1.63 ± 0.20	1.52 ± 0.25
TBC1D1 (AU)	0.25 ± 0.03	0.42 ± 0.06 *	1.47 ± 0.18	1.06 ± 0.18

Data are expressed as mean ± SEM

*P < 0.05 vs. WT mice; **P < 0.001 vs. WT mice; ***P < 0.0001 vs. WT mice

Phosphorylation of Akt substrate of 160 kDa (AS160) on serine site 666 was significantly elevated in the gastrocnemius (72%, *P* < 0.05, Table 2) and phosphorylation of TBC1 domain family member 1 (TBC1D1) on Thr590 was significantly decreased in the soleus (− 31%, *P* < 0.05, Table 2) of bGH compared to WT mice. Phosphorylation on AS160 site Ser588 and TBC1D1 site Ser700 did not differ between the two groups of mice. Total AS160 and TBC1D1 were higher in the gastrocnemius of bGH compared to WT mice (48 and 65%, respectively, *P* < 0.05, Table 2) but did not differ in the soleus. When individual phosphorylation sites were normalized to respective total protein, no differences were detected for AS160 or TBC1D1.

## Discussion

The main finding in the current study was that chronic elevations in the GH/IGF axis regulate critical signaling pathways involved in both skeletal muscle protein synthesis and atrophy. Transgenic bGH mice have elevations in Akt1 expression and increased phosphorylation of downstream targets responsible for muscle hypertrophy. However, these mice also show increased intramuscular signaling typically associated with skeletal muscle atrophy. We provide novel evidence that MuRF1 is upregulated in a muscle group-dependent manner in bGH mice. We also demonstrate for the first time that myostatin, its receptor (AcvR2B), and part of its canonical pathway (Smad2) are upregulated in the skeletal muscle of mice overexpressing GH. Taken together, our findings suggest that in environments of chronic GH and/or IGF-1 excess, MuRF1 and myostatin may act to inhibit excess muscle growth, especially in skeletal muscle containing a high proportion of fast twitch fibers.

To our knowledge, this is the first study to provide evidence that GH regulates Akt in an isoform dependent manner (Fig. 2). One of the primary mechanisms by which GH/IGF stimulates muscle growth is through the IRS/Akt1/mTOR pathway; therefore, it was not surprising that total Akt1 was upregulated in both the gastrocnemius and soleus of bGH mice (Fig. 2a, b). The current study also provides novel evidence that protein synthesis pathways downstream of Akt1, including the 4E-BP1 and p70 kinase pathways are increased in the skeletal muscle of bGH mice (Fig. 3c–f). Protein synthesis involves a complex series of events starting with the translation initiation step and the assembly of the eIF4G-eIF4E complex [46]. In the unphosphorylated form, 4E-BP1 binds to the eukaryotic translation initiation factor 4E (eIF4E) protein, inhibiting it from binding to the extracellular signal-regulated kinase (eIF4G) subunit and consequently repressing translation initiation. Hyperphosphorylation of 4E-BP1, similar to that observed in bGH mice (Fig. 3c), allows 4E-BP1 to dissociate from eIF4E, increasing the availability of eIF4E to bind to eIF4G and increasing protein translation. Mice overexpressing GH also demonstrated increased phosphorylation of the p70 S6 kinase (Fig. 3e–f), a protein critical for the activation of downstream targets responsible for the translation of elongation factors and cell growth [47]. While these results clearly provide evidence that aspects of the Akt1 signaling pathway are upregulated in the skeletal muscle of bGH mice, we also provide novel findings demonstrating that portions of this pathway are unaffected (i.e., GSK3β and FOXO1) or even negatively affected, as is the case for the gastrocnemius (i.e., Akt2 and mTOR, Figs. 2c and 3a), which would contradict previous observations in the liver of bGH mice [48–50]. Taken together, our data indicates that portions of the Akt1 pathway, especially in the gastrocnemius, become less sensitive to chronic elevations in GH/IGF compared to other organs, including the liver [39, 48, 49], and could contribute to the inhibition of excess skeletal muscle growth.

Despite not measuring protein synthesis rates in the present study, lean body mass of bGH mice has been reported to plateau by 4 months of age [39]. Of particular interest, we observed a dramatic decrease in the gastrocnemius mass, after normalizing it to total body mass in bGH mice compared to controls (Table 1). This attenuation of muscle growth occurs in spite of plasma GH and IGF-1 concentrations being elevated approximately 400 and 80%, respectively, over controls [51]. In an attempt to examine if chronic elevations of GH/IGF-1 affect signaling pathways responsible for inhibiting muscle growth, we measured MuRF1, as well as, myostatin and its canonical signaling pathway. Myostatin was elevated in both the soleus and gastrocnemius of bGH mice (Fig. 7a–b), whereas MuRF1 increased only in the latter (Fig. 6). Similar muscle specific increases in MuRF1 have been observed in other models of atrophy [52]. Given the dramatic decline in gastrocnemius muscle mass relative to the total body mass in bGH mice, the muscle-specific upregulation of MuRF1 could contribute to the inhibition of growth in this muscle. To our knowledge, this is the first study to report an increase in MuRF1 expression in a model of chronic GH excess. It remains unclear if the gastrocnemius specific upregulation of MuRF1 is a direct or indirect consequence of excess GH. Despite not observing differences in FOXO1, a known regulator of MuRF1, a number of other stimuli including inflammation and TNF-alpha have been reported to increase this protein [52] and could have played a role. Phosphorylation of skeletal muscle JNK, a pathway typically activated by inflammatory cytokines, was dramatically increased in bGH mice (Fig. 4g), and others have reported elevated TNF-alpha levels in mice overexpressing GH [53], which could have played a role in regulating MuRF1 expression.

In contrast to our findings with MuRF1, myostatin was elevated in both the gastrocnemius and soleus muscle of bGH mice. Myostatin is originally synthesized in skeletal muscle in its precursor form (52 kDa) and then subsequently modified to the mature myostatin form (26 kDa) through a series of proteolysis steps. A novel finding in the current study was that STAT5 phosphorylation levels were associated with myostatin precursor levels (Fig. 7g), lending support to previous conclusions that GH post-receptor signaling may play a role in regulating myostatin [54]. Myostatin inhibits skeletal muscle growth by binding to the myostatin receptor (AcvR2B), leading to phosphorylation of Smad2 and Smad3, which then form a heterodimer with Smad4 to translocate to the nucleus to regulate gene transcription. To our knowledge, this is the first study to demonstrate that the myostatin receptor along with Smad2 phosphorylation (Fig. 7c–d) are elevated in a model of GH excess. A somewhat unexpected finding was that Smad3 phosphorylation did not differ between the two groups of mice (Fig. 7e–f). While the regulation and function of Smad2 and Smad3 have primarily been considered analogous, the current study, along with others [55–57], suggests these two proteins may be regulated differently and have independent physiological roles under certain environmental conditions. IGF-1-induced Akt stimulation has recently been shown to inhibit Smad3 phosphorylation in skeletal muscle cells (myoblasts) [58] and has been reported to selectively inhibit Smad3 phosphorylation with no effect on Smad2 phosphorylation in NRP-152 cells [59]. Therefore, it is conceivable that the chronic elevations of IGF-1 in bGH mice blocked the myostatin-induced Smad3 phosphorylation, without having an effect on SMAD2 phosphorylation.

Given the differing effects of GH excess on the two muscle groups with respect to relative muscle mass (Table 1), the role of increased myostatin signaling in bGH remains unclear. However, given the strong negative association between myostatin levels and relative gastrocnemius muscle mass (Fig. 7h), along with the known preferential action of myostatin on type 2 muscle fibers [30, 60, 61], it is conceivable that the catabolic actions of myostatin are more prevalent in the gastrocnemius than soleus during excess GH/IGF-1 to protect this muscle against excess muscle growth. Similar findings have been described in cardiac tissue, where myostatin has been reported to act as a chalone during conditions of elevated IGF-1 [33, 34]. Collectively, these findings suggest that chronic elevations in GH/IGF-1 may stimulate increased MuRF1 and myostatin protein expression as a mechanism to counterbalance excess stimulation of protein synthesis in skeletal muscle and may contribute to the decrease in gastrocnemius mass with respect to other muscle and organs in bGH mice.

The downstream pathway involved in insulin-induced glucose uptake (AS160 and TBC1D1) appears to be relatively unaffected in bGH mice, at least in the fasted state (Table 2). This finding was somewhat unexpected given the elevated insulin levels in bGH mice of this age [39] and could suggest inhibitory mechanism(s) located upstream. Consistent with previous findings in skeletal muscle [37] and liver [40], we observed diminished insulin receptor expression in the skeletal muscle of bGH mice compared to their WT counterparts (Table 2). Previous research has shown enhanced phosphorylation of IRS1 at Ser 612 and Ser 636/639 in the heart of bGH mice [62]; however, we believe this is the first study to report increased skeletal muscle phosphorylation of IRS1 on serine site 307, in a model of GH excess. Based on our findings that the elevated IRS serine phosphorylation occurred only in the gastrocnemius, it is possible this effect is specific to glycolytic muscle. This observation has significant implications since IRS1 serine phosphorylation on site 307 inhibits both insulin and IGF-1 signal transduction and has become synonymous with conditions of insulin/IGF-1 resistance [63–65], muscle atrophy [66], and inflammation [65]. Increased serine phosphorylation of IRS1 could provide a novel mechanism to explain the insulin resistance reported in bGH mice [37, 40, 41] and could play a role in inhibiting excess IGF-1 signaling in the gastrocnemius. Since both insulin and IGF-1 are known to increase serine phosphorylation of IRS1, it is not unrealistic to suggest that the chronic, elevated levels of these hormones in bGH mice stimulates the serine phosphorylation of IRS1 as another means to counter regulate excess IGF1/Akt1 signaling and prevent excess muscle growth.

The MAPK pathway (ERK and p38) is another pathway stimulated by GH and IGF-1, independent of IRS/Akt, and is critical for the growth and maintenance of skeletal muscle. Of particular interest, the MAPK response to excess GH/IGF-1 appeared to be muscle group specific, with increased phosphorylation of p42/44 and p38 only occurring in the gastrocnemius of bGH mice. The increased protein content of p42 and p38 likely contributed, at least in part, to the increased phosphorylation levels of the proteins. Research investigating the effects of chronic GH overexpression on the liver have reported the upregulation of both p42/p44 protein [48, 67] and somewhat surprisingly, decrements in p38 phosphorylation [68]. While the mechanism responsible for these conflicting findings remain unknown, it could be related to the tissue-specific role of this pathway in muscle cell differentiation [19] and transcriptional regulation of muscle-specific genes [69].

It has previously been determined that the gastrocnemius of mice consists of ~ 3% type I fibers and 55–80% type IIb fibers, whereas the soleus consists of 30–40% type I and ~ 3% type IIb [70, 71] allowing us to examine the effects of chronic GH/IGF-I in separate muscle groups that contain two differing muscle fiber type composition. Our finding that bGH mice had a higher proportion of MHC I protein in both their gastrocnemius (Fig. 8a) and soleus (Fig. 8b) is in line with previous research in bGH mice [42]. The current study did not measure muscle fiber cross-sectional area (CSA), but others have reported that an increase in muscle fiber CSA is primarily in oxidative muscle fibers (type I and IIA), without any significant changes in glycolytic type IIb fibers [42]. GH is known to have metabolic properties promoting lipid oxidation and sparing glucose oxidation [72] which could stimulate the transition of the muscle to a more oxidative fiber type. The cellular mechanism(s) responsible for the preferential switch to more oxidative muscle fibers during GH excess remains unclear; however, a unique finding in the current study was the upregulation of myostatin in muscle demonstrating an increase in MHC I. In addition to the well-documented regulation of muscle mass, myostatin also plays an important role in determining skeletal muscle fiber composition [30]. Animal models deficient in myostatin have increased muscle mass, with a specific increase in fast glycolytic muscle [30, 60, 61]. Therefore, in addition to the potential role in inhibiting excess muscle growth, the increased myostatin levels could have contributed, at least in part, to the increase in MHC I in both the soleus and gastrocnemius of bGH mice.

## Conclusions

The current study demonstrates for the first time the upregulation of a number of cellular mechanisms involved in both skeletal muscle hypertrophy and atrophy under conditions of excess GH/IGF-1. This study also highlights the muscle specific increases in MuRF1 and IRS1 serine phosphorylation that parallel the more dramatic inhibition of muscle growth in the gastrocnemius compared to the soleus of bGH mice. We also provide insight into the elevation of myostatin and MHC I in bGH mice, regardless of muscle type. Taken together, the current study provides critical insight into the signaling pathways involved in regulating skeletal muscle mass under conditions of excess GH/IGF and highlights the importance of fiber type specific studies when investigating the effects of GH on skeletal muscle signaling and muscle growth.

## Abbreviations

4E-BP1: 4E-binding protein 1
A.U.: Arbitrary unit
AcvR2B: Activin receptor type-2B
AIDS: Acquired immune deficiency syndrome
Akt: Protein kinase b
AS160: Akt substrate of 160 kDa
bGH: Bovine growth hormone
CO_2_: Carbon dioxide
COPD: Chronic obstructive pulmonary disease
eIF4E: Eukaryotic translation initiation factor 4E
eIF4G: Eukaryotic translation initiation factor 4G
ERK: Extracellular signal-regulated kinase
FOX: Forkhead box
GAPDH: Glyceraldehyde 3-phosphate dehydrogenase
Gast: Gastrocnemius
GH: Growth hormone
GSK3: Glycogen synthase kinase
IGF: Insulin-like growth factor
IRS1: Insulin receptor substrate 1
JAK2: Janus kinase 2
JNK: c-Jun N-terminal kinase
MAPK: Mitogen-activated protein kinase
MHC: Myosin heavy chain
mTOR: Mechanistic target of rapamycin
MuRF1: Muscle RING finger 1;
NT: Non-transgenic
p70 S6 Kinase (Thr389): Ribosomal protein S6 kinase
PCR: Polymerase chain reaction
SDS-PAGE: Sodium dodecyl sulfate polyacrylamide gel electrophoresis
SEM: Standard error of mean
Ser: Serine
SMAD: Mothers against decapentaplegic homolog
Sol: Soleus
Stat: Signal transducer and activator of transcription
TBC1D1: TBC1 domain family member 1
TGF-β: Transforming growth factor-β
Thr: Threonine
Tyr: Tyrosine
WT: Wild type
